# Risk and cause of death in post-traumatic epilepsy: a register-based retrospective cohort study

**DOI:** 10.1007/s00415-022-11279-5

**Published:** 2022-07-19

**Authors:** Markus Karlander, Johan Ljungqvist, Ann Sörbo, Johan Zelano

**Affiliations:** 1 Department of Clinical Neuroscience, Institute of Neuroscience and Physiology, Sahlgrenska Academy, Gothenburg University, Gothenburg, Sweden; 2 Department of Neurology and Rehabilitation, Södra Älvsborg Hospital, Borås, Sweden; 3 Department of Research, Education and Innovation, Region Västra Götaland, Södra Älvsborg Hospital, Borås, Sweden; 4 Department of Neurosurgery, Sahlgrenska University Hospital, Gothenburg, Sweden; 5 Department of Neurological Care, Sahlgrenska University Hospital, Gothenburg, Sweden; 6 Wallenberg Center of Molecular and Translational Medicine, Gothenburg University, Gothenburg, Sweden; 7 Department of Neurology, Sahlgrenska University Hospital, Blå stråket 7, 413 45 Gothenburg, Sweden

**Keywords:** Epilepsy, Trauma, Epidemiology, Mortality

## Abstract

**Objective:**

Post-traumatic epilepsy (PTE) is common, but its impact on survival after traumatic brain injury (TBI) of different severity and in different demographic patient groups is unknown. We analyzed the risk of death associated with PTE with adjustment for TBI severity, causes of death, and the contribution of epilepsy as direct or contributing cause of death.

**Methods:**

Register-based, retrospective cohort study. All individuals hospitalized in Sweden for a TBI between 2000 and 2010 without prior seizures were identified in the National Patient Register, with follow-up until 2017. Subsequent epilepsy was identified by ICD-10 codes. Time-dependent Cox proportional hazard ratio (HR) was used to assess hazard of death, with epilepsy as a time-updated covariate. Adjusted analyses for age, gender, injury severity and comorbidities were also performed. Causes of death were analyzed using the Cause of Death Register.

**Results:**

Among 111 947 individuals with TBI, subsequent epilepsy diagnosis was associated with a crude HR of 2.3 (95% CI: 2.2–2.4) for death. Stratified analyses showed a HR of 7.8 (95% CI: 6.5–9.4) for death in younger individuals. Epilepsy was a more common underlying cause of death in younger individuals.

**Conclusion:**

PTE is associated with a higher risk of death and epilepsy seems to contribute to a significant proportion of deaths, especially in younger age groups. Future studies on whether improved epilepsy treatment can reduce mortality are needed.

## Introduction

Traumatic brain injury (TBI) is a common cause of epilepsy. Acquired epilepsy in general increases mortality to a median standardized mortality ratio of 2.3 [1]. In post-traumatic epilepsy (PTE), mortality varies in the literature. A US study found an almost three times increased mortality in PTE and an increased risk of death in younger age groups [2]. A Taiwanese study reported a twofold increased mortality in PTE, also after adjustment for age, sex, and comorbidities [3]. Similarly, a Finnish study found 1.75 times higher mortality in PTE after non-mild TBI, but whether PTE or initial TBI severity explained the excess mortality could not be addressed for sample size reasons [4]. This is a key question. If PTE is independently associated with a higher risk of death, studies are needed on possible mechanisms.

We used Swedish register data to investigate the risk of death in PTE, with adjustment for injury severity, in a population-based study including over 100 000 individuals hospitalized for TBI in Sweden between 2000 and 2010. We also analyzed to what extent epilepsy itself was stated as a direct or contributing cause of death.

## Materials and methods

The study was conducted on data from comprehensive Swedish national registers, based on the personal identification number, unique to all inhabitants in Sweden. The National Patient Register (NPR), the Cause of Death Register (CDR) are managed by the National Board of Health and Welfare and the population register is managed by Statistics Sweden. Reporting to the NPR and CDR is mandatory for all health care providers. Information on all hospital inpatient care since 1987 and specialized outpatient care since 2001, with improvements in coverage until 2005 is available through the NPR. Data from the cause of death register identify an underlying cause of death and contributing causes of death (other diseases and injuries contributing to death).

**Fig. 1:**
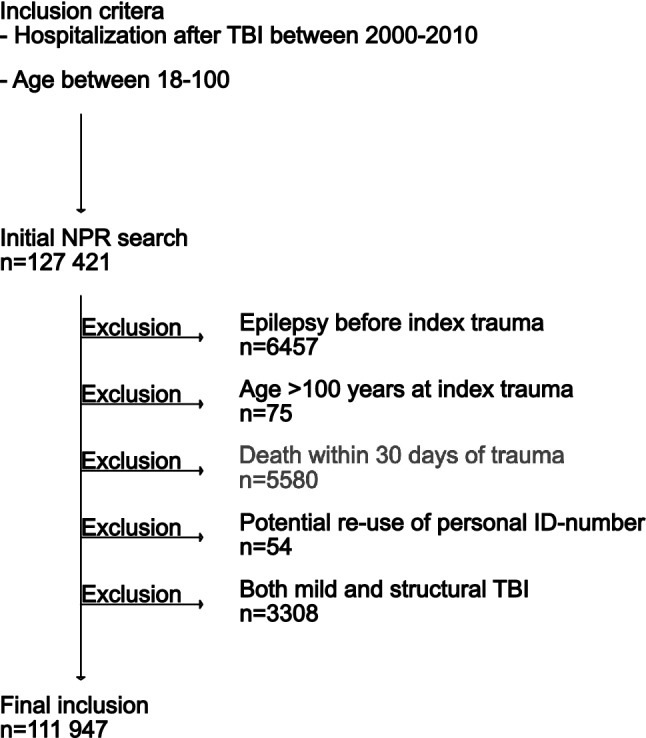
Study inclusion

All individuals aged ≥ 18 hospitalized for a traumatic brain injury (TBI) between 2000 and 2010 were included (Emergency room visits were not included). TBI was defined according to International Classification of Disease (ICD)-10 as a diagnostic code of S06, S02.0-02.1, S02.7, or S02.9 (*n* = 111,947). The inclusion criteria are described in detail in Fig. 1.

### Definitions

Information on trauma, epilepsy, and comorbidities was obtained from the NPR. The index trauma was categorized by ICD-10 codes into mild injury (S060), extracerebral injury (S064, S065, S066), focal cerebral injury (S063), diffuse cerebral injury (S061, S062, S067, S068, S069), or fracture (S020, S021, S027, S029). In the case of several codes, participants were categorized according to the code inferring highest epilepsy risk, as previously described [5].

PTE was defined as the occurrence of ICD-9 code 345 except 345Q, or ICD-10 G40, meeting the definition of probable epilepsy recommended by the commissioned task force of the International League against Epilepsy [6]. Seizure was defined as occurrence of ICD-9 780D or ICD-10 R568. Status epilepticus was defined as occurrence of ICD-9 345Q or ICD-10 G41. CNS (central nervous system) comorbidities were defined by occurrence relevant codes; stroke (ICD-9 430–436, or ICD-10 I60–I64), CNS-tumor (ICD-9 191 225 198D 198E 237F 239G ICD-10: C71 C793 D430 D32 D330), and CNS-infections (ICD-9 006F, 013, 036A, 036B, 045–049, 052B, 053A, 053B, 054D, 054H, 055A, 056A, 062–064, 072B, 072C, 094, 136C, 320–325, ICD-10 A06.6, A17, A39, A80–A89, B00.3, B00.4, B01.0, B01.1, B02.0, B02.1, B05.0, B05.1, B06.0, B22.0, B26.1, B26.2, B37.5, B38.4, B43.1, B50.0, B58.2, B60.2, G00–G08, R29.1).

Date of death and cause of death was obtained from the CDR based on ICD-10 codes as follows: ischemic heart disease (IHD) (I2), cerebrovascular disease (CVD) (I6), other heart disease (I3–I5, I0), other vessel disease (I1, I7, I8, I9), infectious diseases (A00–B99), endocrine, nutritional and metabolic diseases (E00–E90), mental and behavioural diseases (F00–F99), disorders of the nervous system (G00–G99), malignancy (C00–D48), diseases of the respiratory system (J00–J99), diseases of the digestive system (K00–K93), external cause of mortality (V01–Y98), sequelae of external causes of mortality (Y85–Y89) and other (D50–D89, H00–H59, L00–L99, M00–M99, N00–N99, P00–P96 Q00–Q99, R00–R99).

### Statistical analyses

All analyses were performed in SPSS version 26 (IBM, NY, USA), except for the time-dependent multivariable Cox proportional regression modeling and Consecutive Cox models which were done in SAS, version 9.4 (Cary, NC, USA).

HR of death for the entire population and different subgroups was calculated by time-dependent Cox proportional regression modeling with admission date for TBI as start, death as event, and censoring at death or 2017-12-31, either with just PTE entered as a time-dependent covariate (univariate), or with multivariable adjustment for age, sex, type of injury, and time-updated CNS comorbidities. Consecutive Cox models were performed dividing the follow-up time into 6 months periods, to validate the assumptions of the Cox proportional regression modeling.

Significance analyses were performed using chi-squared test for Cox proportional regression model and *z* test for causes of death.

### Sensitivity analyses

In one sensitivity analysis, individuals with stroke, CNS-tumors, and CNS-infection were excluded—leaving 88 584 individuals for analysis. In another sensitivity analysis, only individuals with a first epilepsy diagnosis within 2 years of index date (the typical latency of PTE) [5], were classified as PTE (*n* = 2060).

## Results

### PTE vs non-PTE

The median age at the time of trauma was slightly higher in the PTE group than in the non-PTE group (58, range 18–99 vs 56, range 18–100). Male sex and brain comorbidities were more common in PTE. Individuals with PTE had in general a more severe TBI, although mild injury was most common in both groups (Table 1).

**Table 1:** Demographics, type of injury, comorbidities, and death by exposure

	Post-traumatic epilepsy		No post-traumatic epilepsy	
	*n* = 4292		*n* = 107 655	
	*n*	% (95% CI)	*n*	% (95% CI)
Age index				
18–39	921	21.5 (20.2–22.7)	34 770	32.3 (32.0–32.6)
40–59	1390	32.4 (31.0–33.8)	23 492	21.8 (21.6–22.1)
60–79	1439	33.5 (32.1–35.0)	26 108	24.3 (24.0–24.5)
79<	542	12.6 (11.7–13.6)	23 285	21.6 (21.4–21.9)
Gender				
Male	2756	64.2 (62.8–65.6)	62 877	58.4 (58.1–58.7)
Female	1536	35.8 (34.4–37.2)	44 778	41.6 (41.3–41.9)
Type of injury				
Mild	1991	46.4 (44.9–47.9)	74 338	69.1 (68.8–69.3)
Fracture	181	4.2 (3.6–4.8)	5900	5.5 (5.3–5.6)
Extracerebral	967	22.5 (21.3–23.8)	15 525	14.4 (14.2–14.6)
Diffuse cerebral	641	14.9 (13.9–16.0)	8137	7.6 (7.4–7.7)
Focal cerebral	512	11.9 (11.0–12.9)	3755	3.5 (3.4–3.6)
Comorbidities				
Stroke	1705	39.7 (38.3–41.2)	19 724	18.3 (18.1–18.6)
CNS-tumor	224	5.2 (4.6–5.9)	1209	1.1 (1.1–1.2)
CNS-infection	173	4.0 (3.5–4.7)	1142	1.1 (1.0–1.1)

The median time from injury to epilepsy was 2 years (range: 0–18 years) and median time from trauma to death was 6.31 years in the PTE group and 3.92 years in the non-PTE group.

### Survival

During follow-up, 48.1% (95% CI: 46.6–49.6) in the PTE group died, compared to 37.6% (95% CI: 37.3–37.9) in the non-PTE group. The incidence proportion of death was highest during the first year after the epilepsy diagnosis in the PTE group, in which 27.1% (95% CI: 25.2–29.0) of the total deaths occurred.

### All-cause mortality

All-cause mortality from the time of epilepsy was 77.3 deaths/1000 person-years in the PTE group. When stratified by injury severity, the all-cause mortality was 63.0 deaths/1000 person-years after focal cerebral injury, 64.4 deaths/1000 person-years after diffuse cerebral injury, 100.8 deaths/1000 person-years after extracerebral injury, 65.7 deaths/1000 person-years after fracture and 76.7 deaths per 1000 person-years after mild injury.

### Hazard ratio for death

The HR of death was doubled in patients with PTE, and only slightly affected by adjustment for age, sex, type of injury, and comorbidities. Stratified analyses showed that the association between PTE and death was significantly higher in younger age groups, with an eightfold hazard of death in individuals 18–39 years of age (Table 2).

**Table 2:** Cox proportional hazard ratio of death risk after PTE compared to non-PTE

	Crude HR	Significance level (*p* value)	Adjusted HR	Significance level (*p* value)
All	2.3 (95%CI:2.2–2.4)	< 0.001	1.8 (95%CI: 1.8–1.9)	< 0.001
Subgroups				
Type of injury				
Mild	2.9 (95%CI:2.7–3.1)	< 0.001	2.1 (95%CI: 1.9–2.2)	< 0.001
Fracture	2.2 (95%CI:1.7–2.8)	< 0.001	1.5 (95%CI: 1.2–1.9)	< 0.001
Extracerebral	1.3 (95%CI:1.2–1.4)	< 0.001	1.6 (95%CI: 1.5–1.7)	< 0.001
Diffuse cerebral	1.6 (95%CI:1.4–1.8)	< 0.001	1.7 (95%CI: 1.5–2.0)	< 0.001
Focal cerebral	1.6 (95%CI:1.4–1.8)	< 0.001	1.7 (95%CI: 1.5–2.0)	< 0.001
Age at TBI				
18–39	7.8 (95%CI: 6.5–9.4)	< 0.001	4.9 (95%CI: 3.9–6.0)	< 0.001
40–59	4.0 (95%CI: 3.6–4.3)	< 0.001	2.6 (95%CI: 2.3–2.8)	< 0.001
60–79	2.1 (95%CI: 2.0–2.2)	< 0.001	1.8 (95%CI: 1.6–1.9)	< 0.001
79+	1.3 (95%CI: 1.2–1.4)	< 0.001	1.3 (95%CI: 1.2–1.5)	< 0.001
Sex				
Male	2.6 (95%CI: 2.4–2.7)	< 0.001	1.9 (95%CI: 1.8–2.0)	< 0.001
Female	2.0 (95%CI: 1.9–2.2)	< 0.001	1.8 (95%CI: 1.7–1.9)	< 0.001

Crude, univariate HR after PTE; Adjusted HR, with adjustment for age (continuous), sex, type of injury, and comorbidities

There was no significant difference between the adjusted HR of death in men compared to women, or between different types of structural TBI. When adjusting for age, sex, type of injury, and comorbidities, the HR was still increased in patients with PTE in all patient strata.

### Cause of death

We next compared registered causes of death between individuals with PTE and controls. Cerebrovascular disorders, disorders of the nervous system, disorders of the digestive system, and external causes (including trauma) were more common causes of death in individuals with PTE (Table 3).

**Table 3:** Causes of death in PTE and non-PTE

ICD chapter	PTE		Non-PTE		Significance (*p* value)
	*n*	% (95% CI)	*n*	% (95% CI)	
Ischemic heart disease	255	12.4 (11.0–13.8)	6720	16.6 (16.3–17.0)	< 0.001
Cerebrovascular disease	305	14.8 (13.3–16.4)	4254	10.5 (10.2–10.8)	< 0.001
Other heart disease	152	7.4 (6.3–8.6)	4191	10.4 (10.1–10.7)	< 0.001
Other vessel disease	79	3.8 (3.1–4.7)	1825	4.5 (4.3–4.7)	Not significant
Infectious diseases	47	2.3 (1.7–3.0)	893	2.2 (2.1–2.4)	Not significant
Endocrine, nutritional, and metabolic disease	59	2.9 (2.2–3.6)	1051	2.6 (2.4–2.8)	Not significant
Mental and behavioural diseases	168	8.1 (7.0–9.4)	3804	9.4 (9.1–9.7)	Not significant
Disorders of the nervous system	131	6.3 (5.4–7.5)	1870	4.6 (4.4–4.8)	< 0.001
Malignancy	293	14.2 (12.7–15.8)	5953	14.7 (14.4–15.1)	Not significant
Diseases of the respiratory system	124	6.0 (5.0–7.1)	2552	6.3 (6.1–6.5)	Not significant
Disorders of the digestive system	100	4.8 (4.0–5.8)	1410	3.5 (3.3–3.7)	0.001
External cause of mortality	259	12.6 (11.2–14.0)	3482	8.6 (8.3–8.9)	< 0.001
Other	91	4.4 (3.6–5.4)	2443	6.0 (5.8–6.3)	0.002

**Fig. 2:**
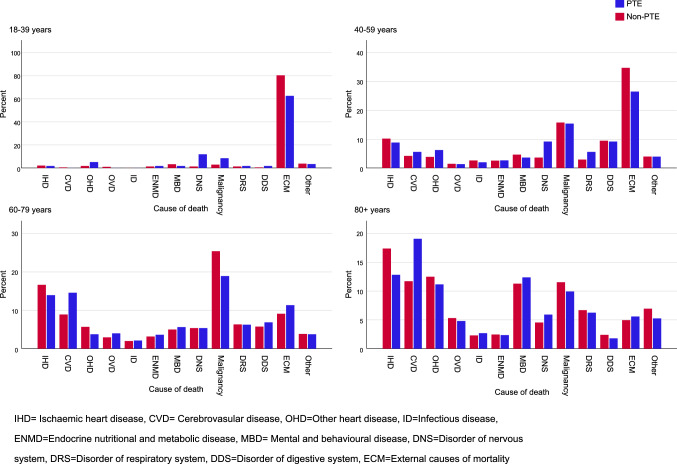
Causes of death in exposed group compared to unexposed, stratified by age groups at time of death. *IHD* ischemic heart disease, *CVD* cerebrovascular disease, *OHD* other heart disease, *ID* infectious diseases, *ENMD* endocrine nutritional and metabolic disease, *MBD* mental and behavioural diseases, *DNS* disorder of nervous system, *DRS* disorder of respiratory system, *DDS* disorder of digestive system, *ECM* external cause of mortality

In individuals between 18 and 59, disorders of the nervous system (including epilepsy) were significantly more common as underlying cause of death in the PTE group (9.6%, 95% CI: 6.9–13.0 vs 3.1%, 95% CI: 2.5–3.8) (Fig. 2).

### Death associated with epilepsy

**Fig. 3:**
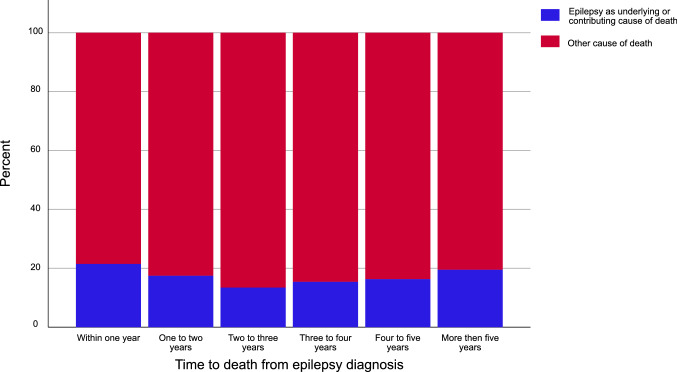
Epilepsy as underlying or contributing cause of death from time of epilepsy diagnosis

Epilepsy, including seizure and status epilepticus, was an underlying or contributing cause of death in 18.4% (95% CI: 16.8–20.1) and the underlying cause in 2.3% (95% CI: 1.7–3.0%), of the individuals with PTE. The proportion of deaths with epilepsy as underlying or contributing cause was stable over the first five years from the diagnosis. (Fig. 3). In the PTE group, epilepsy or status epilepticus was a significantly more common cause of death in individuals dying < 60 years of age, compared to the two older age groups.

### Sensitivity analyses

Since PTE typically develops within 2 years of TBI [5], we performed a sensitivity analysis including only individuals with epilepsy diagnosed within 2 years of their injury. In this group, the all-cause mortality from the time of epilepsy diagnosis was 69.6 per 1000 patient years in this group and the HR for death was 1.9 (95%CI: 1.7–2.0). Another sensitivity analysis assessing the HR for death excluding individuals with a diagnosis for stroke, CNS-infection, and CNS-tumors was performed, showing no significant difference from the main analysis—2.2 (95%CI: 2.1–2.4).

## Discussion

We found a twofold increased risk of death after PTE, and the risk remained elevated with adjustment for, sex, age, type of injury, and comorbidities. To our knowledge, this is the largest study so far evaluating risk of death in PTE and one of the first with a data set allowing adjustment for TBI severity. The elevated HR associated with PTE was highest in younger individuals.

The fact that PTE in itself was associated with increased risk of death, and not merely a representation of TBI severity, highlighting a need for more studies on mechanisms and preventive measures. The findings are probably generalizable; the rate of death in our material was similar to that seen in a recent study on PTE [3], which is in turn substantially higher than after TBI in general [7].

Epilepsy, seizure, or status epilepticus was stated as an underlying or contributing cause of death in 18,4% of patients with PTE, with epilepsy being a more common cause of death in younger individuals.

That the association between death and PTE was highest in younger age groups is a cause for concern. External causes of death were significantly more common in PTE, iterating previous reports that accidents are overrepresented in adults with epilepsy [8]. Sudden unexpected death in epilepsy is another common cause of death among younger adults with epilepsy [9], suicide is also more common among individuals with epilepsy and those after TBI [10, 11]. Our findings indicate that the number of life years lost to epilepsy-related deaths in PTE could be substantial. It is probably premature to conclude that PTE-related deaths are more common in young individuals. Older individuals are at higher risk of dying in general, which could have attenuated the effect of PTE-associated mortality in our regression models.

PTE carried an increased adjusted HR for death to a similar degree in both males and females, This is in line with previous findings in PTE [3] and suggests that although males have a higher standardized mortality ratio among epilepsy patients in general [1], this may not extend to PTE.

Furthermore, the adjusted increased HR for death associated with PTE was similar in different trauma severity groups, indicating that the effect of PTE is proportional to the trauma severity.

Our study was register-based, with associated strengths and weaknesses. One strength is that NPR-registration in Sweden is mandatory in specialized health care, and an ICD-code for epilepsy has > 90 percent accuracy and the register in general an accuracy of 85–95% [12, 13]. Another strength is the cohort size and population-wide design, which increases generalizability, at least to high-income countries with a similar panorama of injury mechanisms for TBI. The levels of PTE and death closely match those described in the literature [3]. There are also limitations. Since the study was register based, we are unable to assess clinical signs related to the trauma which can affect epileptogenesis, such as location of trauma lesion, length of unconsciousness or amnesia [14]. Another limitation is that individuals classified as PTE may have epilepsy for other reasons than TBI, to address this issue a sensitivity analyses was performed, including only patients that received the epilepsy diagnosis within two years after TBI—the typical latency of PTE [5]—showing only a slightly lower HR, and a similar all-cause mortality (Table 2). Another analysis assessing possible contribution of other comorbidities as a cause of the epilepsy, we excluded individuals with stroke, CNS-tumor, and CNS-infection, which did not alter our main results. Since epilepsy is generally under-recognized as cause of death, findings on causes of death should be interpreted with some caution.

Our findings raise important questions. Can improved epilepsy management reduce mortality? Better information on seizure-related risks could perhaps prevent or mitigate accidents. Since many patients with PTE were older and cerebrovascular disease was an important cause of death, another question is whether use of enzyme-inducing anti-seizure medications like phenytoin or carbamazepine may have interfered with cardiovascular primary or secondary prophylaxis.

In conclusion, we found an increased HR of death in individuals with PTE, also with adjustment for TBI severity. The HR was mainly increased in younger individuals. More in-depth study of the healthcare provided to PTE patients are needed to elucidate modifiable mechanisms underlying the higher risk of death.

## Data Availability

The data from the Swedish National Patient Register cannot be shared by the authors because of confidentiality laws.
